# Microstructure and nanoindentation behavior of Cu composites reinforced with graphene nanoplatelets by electroless co-deposition technique

**DOI:** 10.1038/s41598-017-01439-3

**Published:** 2017-05-02

**Authors:** Qi Zhang, Zhenbo Qin, Qin Luo, Zhong Wu, Lei Liu, Bin Shen, Wenbin Hu

**Affiliations:** 1 State Key Laboratory of Metal Matrix Composites, Shanghai Jiao Tong University, Shanghai, 200240 China; 2 Collaborative Innovation Center for Advanced Ship and deep-Sea Exploration, Shanghai, 200240 China; 3 School of Material Science and Engineering, Tianjin University, Tianjin, 300072 China

## Abstract

A reduced graphene oxide/copper (RGO/Cu) composite was fabricated by a surfactant free, electroless co-deposition technique. The graphene oxide (GO) sheets were reduced and RGO homogeneous distributed into the copper matrix. On the basis of nanoindentation, the presence of RGO and the increase of its content in matrix significantly raised the hardness of RGO/Cu composites. Here, the relevant strengthening effect and mechanisms involved in RGO-reinforced Cu composites were systematically evaluated. Especially, the addition of RGO in Cu matrix led to the compressive micro-strain, and the resulted distortion of the lattice parameter was calculated based on Cohen’s method. However, excessive addition of GO in the electrolyte could decrease the mechanical performance due to agglomeration of RGO. Apparently, the optimal concentration for GO dispersion in co-deposition solution was deserved to discuss. After a serious of relative experiments, we could get a conclusion that this method provided a new pathway for embedded graphene into the metal matrix to improve the mechanical properties of RGO-reinforced materials.

## Introduction

Metal matrix composites (MMCs) have been widely applied in aerospace, electronic packing and automotive industries^1^. Carbonaceous nanomaterials, as one type of promising reinforcements for MMCs, have attracted considerable interests due to their excellent electrical, thermal and mechanical properties^2^. Among them, graphene of a one-atom-thick and two-dimensional honeycomb lattice, is a hotspot in the last several years. Compared with carbon nanotubes, graphene had relative higher specific surface areas and lower tendency to twist, which made the graphene sheets disperse better in the matrix. Consequently, the mechanical performance of the MMC improved remarkably^3^. Current researches on graphene-reinforced composites were primarily focused on polymer matrix composites (PMCs). In comparison, the applications of graphene were limited in the field of MMCs, which was attributed to the inhomogeneous dispersion of graphene in the metal matrix caused by strong Van der Waals forces between graphene^4–6^. Another challenge was to keep the structure integrity of graphene during severe deformation such as ball-milling^3, 4^.

To date, the conventional powder metallurgy was a widely used technique for fabricating graphene-reinforced metal matrix composites^5, 7–10^. However, in the metallurgical process, it was difficult to disperse graphene uniformly in the matrix due to large density difference between graphene and metal matrix. Besides that, it also faced detrimental interfacial reaction of graphene and metal matrix at high temperature^3^. Therefore, several novel approaches have been developed such as electro-deposition^11, 12^, friction stir processing^13^ and gas tungsten arc^14^. Friction stir processing and gas tungsten arc, as solid state methods, both operated at high temperature with complicated equipment. Electro-deposition required external source of electrical current, and was confined deposit on conductive substrates^15^. Compared with the above technologies, electroless co-deposition was a cost-effective method without expensive equipment, and also there was no limit to the deposit substrates. In fact, electroless deposition is an effetive technique for metallizing insulators such as plastics, glass and objects with irregular structures which are difficult to coat by other methods^16, 17^. Moreover, it was relative easy to disperse graphene oxide (GO) uniformly in liquid environment rather than solid state. Additionally, it is noteworthy that different fabrication methods to synthesis graphene-reinforced composites might cause different microstructure, and the lattice parameter variation depended on the particle size and the particle shape^18, 19^. Therefore, the distortion of the metal lattice caused by incorporation of RGO into the Cu matrix varied by fabrication approaches. However, nowadays no investigations studied the effect of two-dimensional platelet reinforcement on the lattice parameter of metal.

Generally, graphene tend to agglomerate with each other due to their high specific surface area. In previously reports, polymeric surfactants were usually added in order to improve the dispersion ability of graphene, such as CTAB, SDS, PAA5000, etc.^11, 20^. However, most surfactants have negative effect on the performance of composites as well as potential crisis to the environment^21^. In addition, negatively charged GO could combined with metallic ions through electrostatic attractions and form agglomerates. Therefore, in the case of surfactant-free electrolyte, the optimal concentration of GO/metallic cations precursor suspensions should also be found in order to keep metallic cations stably coexist with GO and maximize its mechanical performance^22, 23^.

To the best of our knowledge, no article has been published on the preparation and characterization of graphene-reinforced copper matrix composites by means of electroless co-deposition and investigated its strengthening mechanism. In this paper, a facile method of electroless co-deposition was introduced for the synthesis of bulk RGO/Cu composites. In this way, the metal nanoparticle in the dispersion could function as a spacer, and graphene sheets can be separated by these metal nanoparticles and thus impede the aggregation of graphene^24^. Additionally, mechanical properties of prepared RGO/Cu composites were investigated using nanoindentation, and relevant strengthening mechanisms were discussed. Furthermore, the micro-strain and lattice parameter after incorporation of RGO in Cu matrix were calculated. Since different content of fillers could lead to different performance of nanoindentation, and thus the optimum content of GO in the electrolyte were also studied.

## Results and Discussion

**Figure 1:**
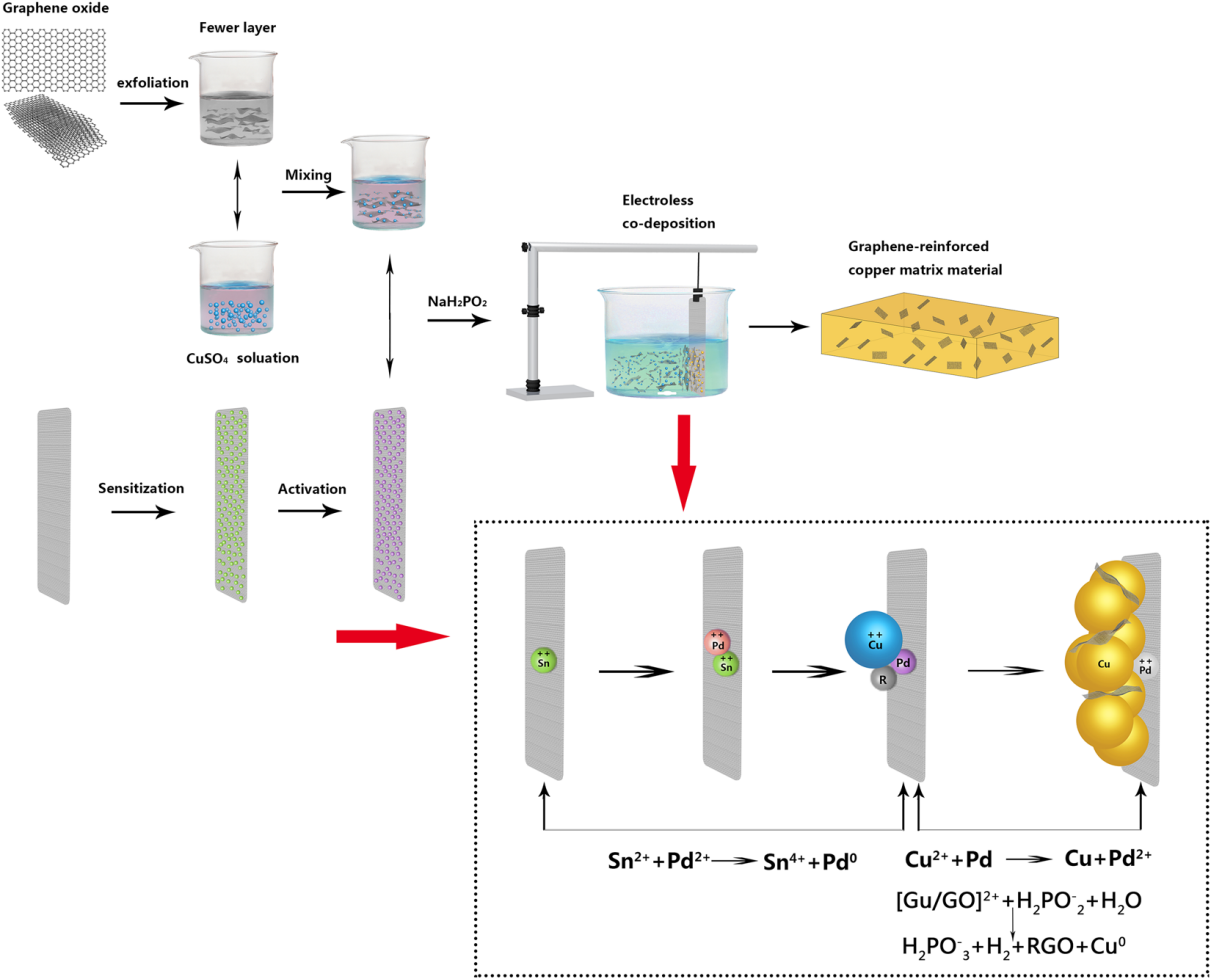
Schematic diagram of the electroless co-deposition technique for preparing graphene-reinforced copper composites.

**Figure 2:**
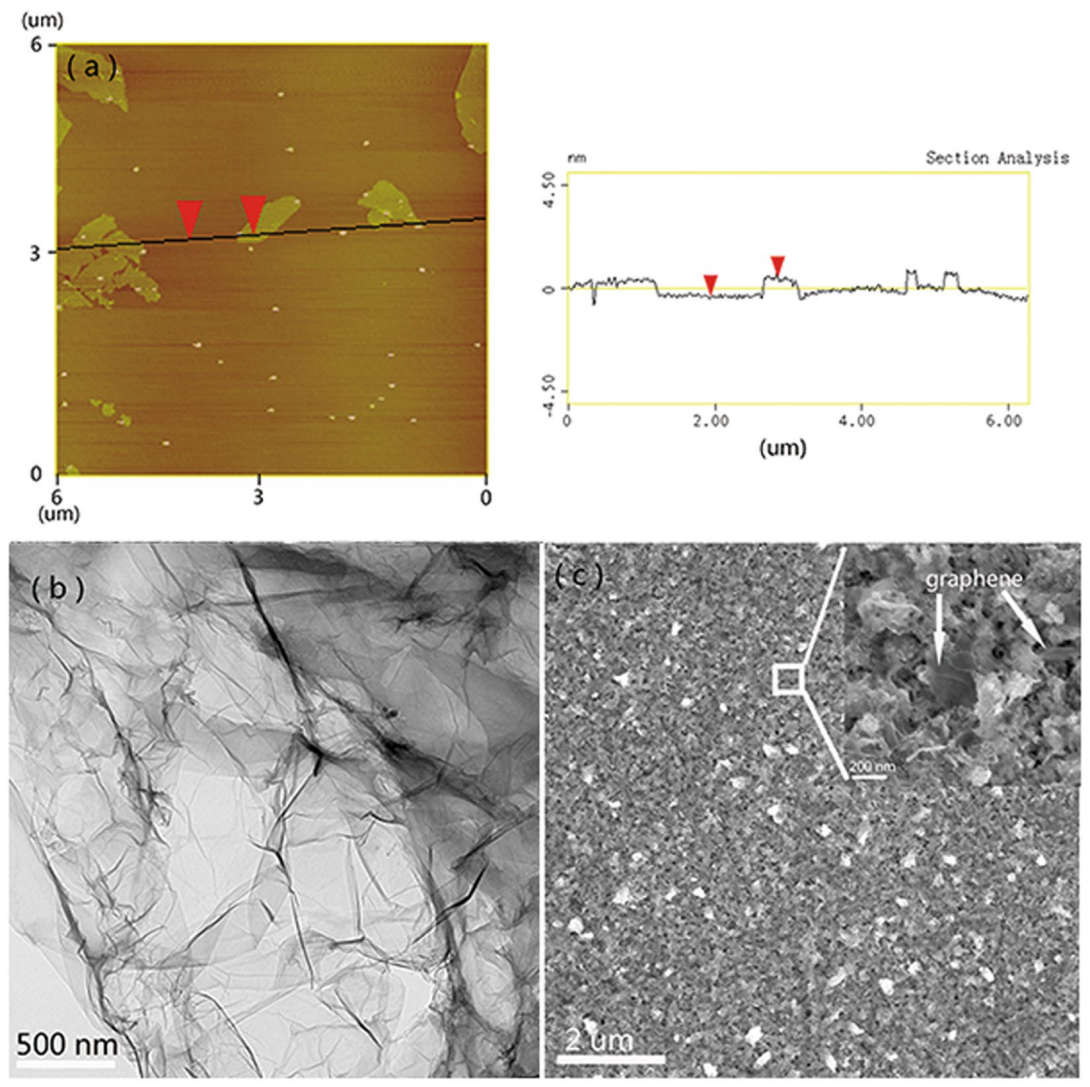
(**a**) AFM image of as-prepared GO sheets deposited onto a mica substrate. The height profile along the black line indicated a sheet thickness of GO was about 0.82 nm. (**b**) TEM image of single layer GO, showing the wrinkled nature of the GO. (**c**) SEM image of RGO/Cu composites after etch at nitric acid solution for 30 seconds. The inset revealed graphene uniformly embedded in the copper matrix (graphene was marked by arrows).

Figure 1 showed the schematic illustrations of the synthesis of RGO/Cu composite by the electroless co-deposition technique. The more detail can be seen in the section of Methods. Figure 2a displayed a typical AFM image of exfoliated GO and the thickness can be clearly identified (0.82 nm), which was consistent with the previous studies^25^. Figure 2b presented a representative TEM image of the GO, showing a paper-like structure with corrugations and ripples. Combined with the AFM results, it suggested that the GO used for co-deposition have been efficiently exfoliated and the few-layer GO dispersion was obtained after sonication. Figure 2c showed the surface SEM image of RGO-reinforced Cu composite, which was obtained after etching at nitric acid solution about 30 seconds. It can be seen from the inset image of Fig. 2c that the graphene sheets were homogeneous dispersed and embedded in the copper matrix (marked by the arrows). Moreover, the SEM micrograph of RGO/Cu composites associated with EDS are shown in Figure S1 (Supporting information).

**Figure 3:**
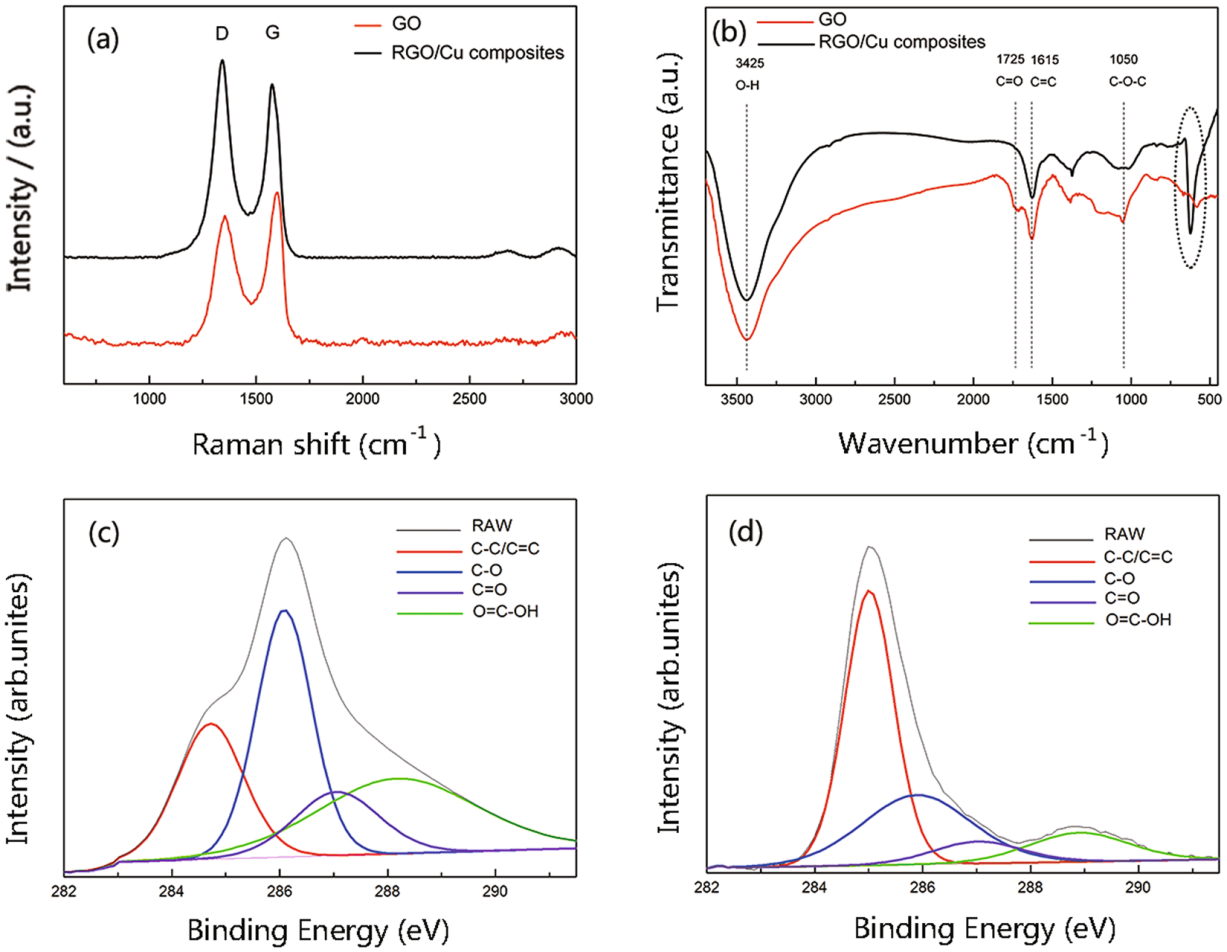
(**a**) Raman spectra and (**b**) FTIR spectrum of as-prepared GO and RGO; Deconvoluted high-resolution C 1 s XPS spectra of (**c**) GO and (**d**) RGO.

In the Raman spectra (Fig. 3a), the G line is assigned to the E_2g_ phonon of C sp^2^ atoms, while the D line is a breathing mode of k-point phonons of A_1g_ symmetry. The D and G band peaks were observed at 1355 cm^−1^ and 1596 cm^−1^ for GO, and then shifted to 1341 cm^−1^ and 1574 cm^−1^ after reduction to RGO/Cu composites. This change was defined as blue shift, which indicated the recovery of the hexagonal network of carbon atom in graphene^26^. For the spectrum of RGO/Cu, the I_D_/I_G_ ratio (1.14) increased compared with that of GO spectrum (0.833), indicating that there was a decrease in the size of the in-plane sp^2^ domains, the removal of the oxygen functional groups in GO and the restoration of the conjugated graphene network after reduction of GO to RGO^27, 28^. Figure 3b showed the FTIR spectra of GO and as-prepared RGO/Cu composites. The characteristic peaks of 3425 cm^−1^, 1725 cm^−1^, 1615 cm^−1^ and 1050 cm^−1^ can be assigned to the O-H stretching vibrations of the C-OH groups, C=O stretching vibrations from carbonyl groups, C=C configurable vibrations from the aromatic zooms and C-O vibrations from alkoxy groups^10^. Compared with the GO, the intensities of the peaks at C=O and C-O for RGO/Cu decreased and C=C increased, indicating that oxygen-containing functional groups of GO have decomposed partially. Therefore, both results of Raman spectroscopy and FTIR provided convincing proof that the GO sheets have been effectively reduced by electroless co-deposition process.

XPS was performed to further characterize the reduction degree of the RGO. In the deconvoluted C 1 s spectrum of GO and RGO/Cu (Fig. 3c,d), four different peaks at 284.9 eV, 286.5 eV, 287.1 eV and 288.9 eV were observed, corresponding to C-C/C=C, C-O, C=O and O=C-OH groups, respectively^29, 30^. After electroless reduction, it can be found that the peak intensities of carbons binding to oxygen significantly decreased, while the peak of C-C/C=C increased accordingly. Specifically, the peak area ratios of the C-O, C=O and O=C-OH bonds to the C-C/C=C of GO were 1.527, 0.593 and 1.325, and the corresponding ratios of RGO were 0.562, 0.145 and 0.211, respectively. This result indicated that the most of oxygen-containing functional groups have been removed and GO sheets were effectively transform to graphene by NaH_2_PO_2_^29, 30^.

**Figure 4:**
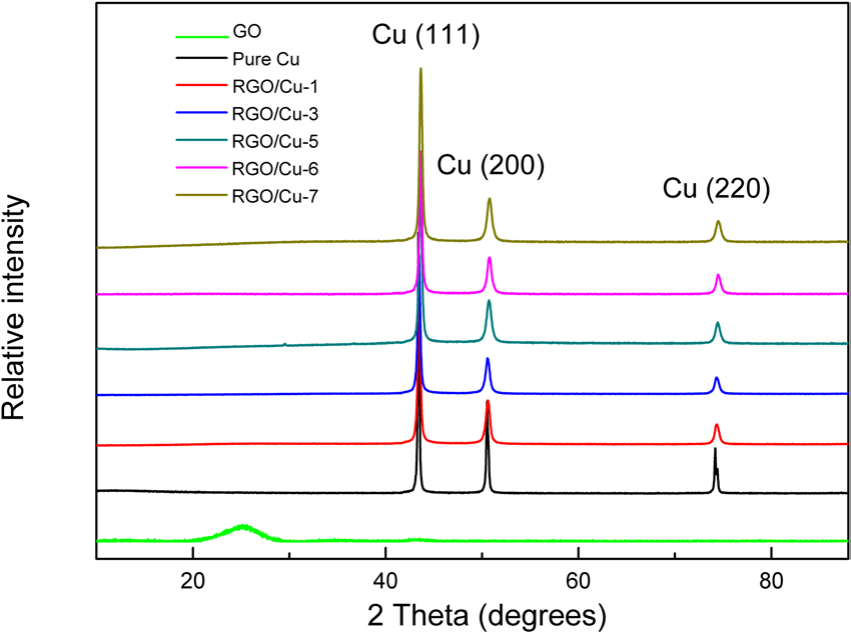
XRD patterns of the RGO, pure Cu and RGO/Cu composites.

Figure 4 showed the XRD patterns of GO and RGO/Cu samples, and the RGO/Cu composites added with different GO contents in the bath (from 0 to 7 mg/L) were prepared and measured (Composites were denoted as RGO/Cu-1, RGO/Cu-3 and RGO/Cu-5, RGO/Cu-6 and RGO/Cu-7, respectively). There was a diffraction peak at 9.6° which correspond to GO (002) plane^31^. Moreover, there were three reflection peaks for RGO/Cu samples located at 43.34°, 50.47° and 74.12°, which can be assigned to Cu (111), Cu (200) and Cu (220), respectively (JCPDS No. 65-9026). Besides that, no additional peaks of copper oxides and graphite for the RGO/Cu composites were identified, revealing that there was no oxides/carbides formation in the synthesis process. Furthermore, the peak of RGO (26°) has not been found in the all RGO/Cu samples which have been reported from other previously studies^32, 33^. This was because the content of RGO in composites was too small to measure.

**Figure 5:**
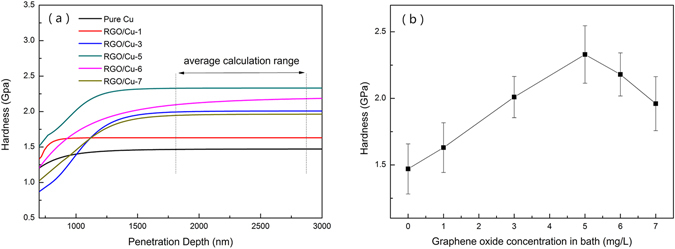
(**a**) Hardness as a function of the penetration depth obtained by CSM method for RGO/Cu composites which containing 0–7 mg/L GO in the electrolyte. The fitting curve was superimposed on this graph and five indentation data for each sample were used in statistical calculations. (**b**) Curve of ultimate hardness of RGO/Cu composites.

Figure S2 showed that the thickness of the as-prepared RGO/Cu composite was nearly 150 um (supporting information for the cross section of the composite). Actually, measuring the hardness of the composite with such a thin thickness is quite challenging. Previous studies demonstrated that nanoindentation technique has been extensively employed for measuring mechanical properties of small volumes of material, e.g. thin film^34, 35^. It was also capable to measure the hardness of materials produced by similar methods, such as Cu-graphene composites foils synthesized by pulse reverse electrodeposition and Graphene/nickel composites prepared by electrodeposition process^11, 12^. Therefore, we determined the hardness of RGO/Cu composites through the nanoindentation technique. Figure 5 showed a typical hardness-displacement curves with respect to RGO/Cu composites by means of CSM technique. Unfortunately, because of indentation size effect, surface roughness and friction between the indenter and sample, there was a scatter in the measured hardness profiles. Therefore, the values below penetration depth of 500 nm in our case were not taken in order to void error during the indentation tests^36^. In our case, the average values in the penetration depth of 2000–2500 nm were accepted as the hardness of the sample due to the minimum variation in this range^34^. The nanohardness values of the RGO/Cu composites with different contents of RGO were listed in Fig. 5b. It can be seen that the hardness of Pure Cu was 1.47 GPa and that of RGO/Cu composites (RGO/Cu-1, RGO/Cu-3, RGO/Cu-5, RGO/Cu-6, RGO/Cu-7) were 1.63, 2.01, 2.33, 2.18 and 1.96 (GPa), respectively. Additionally, five points at each sample were measured to obtain the average value and the hardness of every point was shown in Figure S3.

According to the nanoindentation tests, it was evident that the RGO/Cu composites exhibited higher hardness than that of pure Cu. Apparently, the hardness of the composites increased with the increase of adding GO contents (0–5 mg/L), but then significantly decreased with further increasing the content of GO in the bath. Based on the previous research on carbon reinforcement metal material, the strengthening mechanisms for RGO/Cu composites can be attributed to synergistic effect of grain refinement^2, 6, 14, 37^, load transfer^2, 38, 39^, thermal expansion mismatch^14, 39–41^ and Orowan looping^39, 40^.

**Figure 6:**
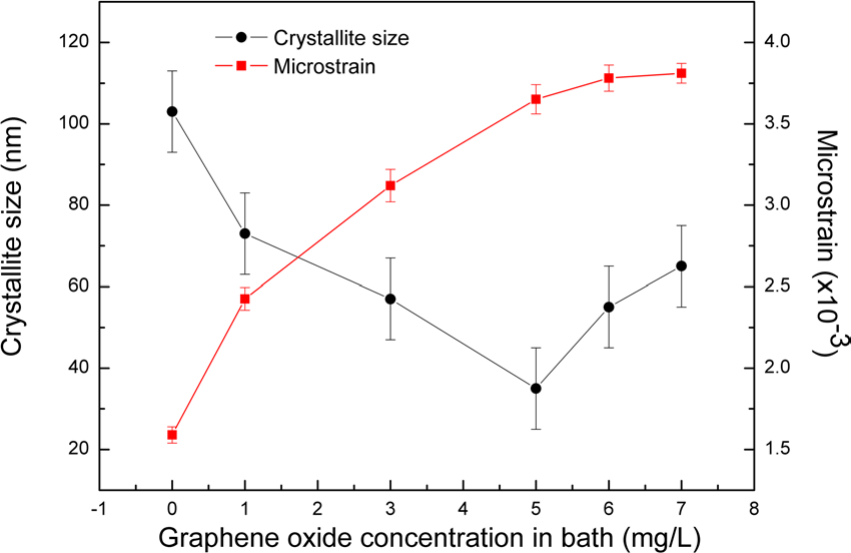
Crystallite size and microstrain of the RGO/Cu composites at different GO loading in bath.

Crystallite size and internal microstrain of RGO/Cu composites were calculated using XRD patterns and summarized in Fig. 6. Compared to the pure Cu sample, intensity of the peak of RGO/Cu composites became lowering and broadening with the increase of the RGO contents. This was due to deformation, grain refinement and straining introduced by the carbon reinforcements^42, 43^. According to the FWHM of Cu (111) peak, the crystallite (D) and microstrain (ε) of RGO/Cu composites could be calculated according to the Voigt function and the Scherrer equation^44^:

1 $${\beta }_{{\rm{C}}}^{h}={\beta }_{C}^{f}+{\beta }_{C}^{g}$$

2 $${\beta }_{G}^{{h}^{2}}={\beta }_{G}^{{f}^{2}}+{\beta }_{G}^{{g}^{2}}$$

3 $$D=\frac{\lambda }{{\beta }_{C}^{f}\,\cos \,\theta }$$

4 $$\varepsilon =\frac{{\beta }_{G}^{f}}{4\,\tan \,\theta }$$

where λ and θ represent the wavelength of Cu-Kα and Bragg angle. The β represents integral breadth. h, f, g represent the measured, structurally broadened and standard line profiles, and subscript C, G represent the Cauchy and Gaussian components, respectively.

**Figure 7:**
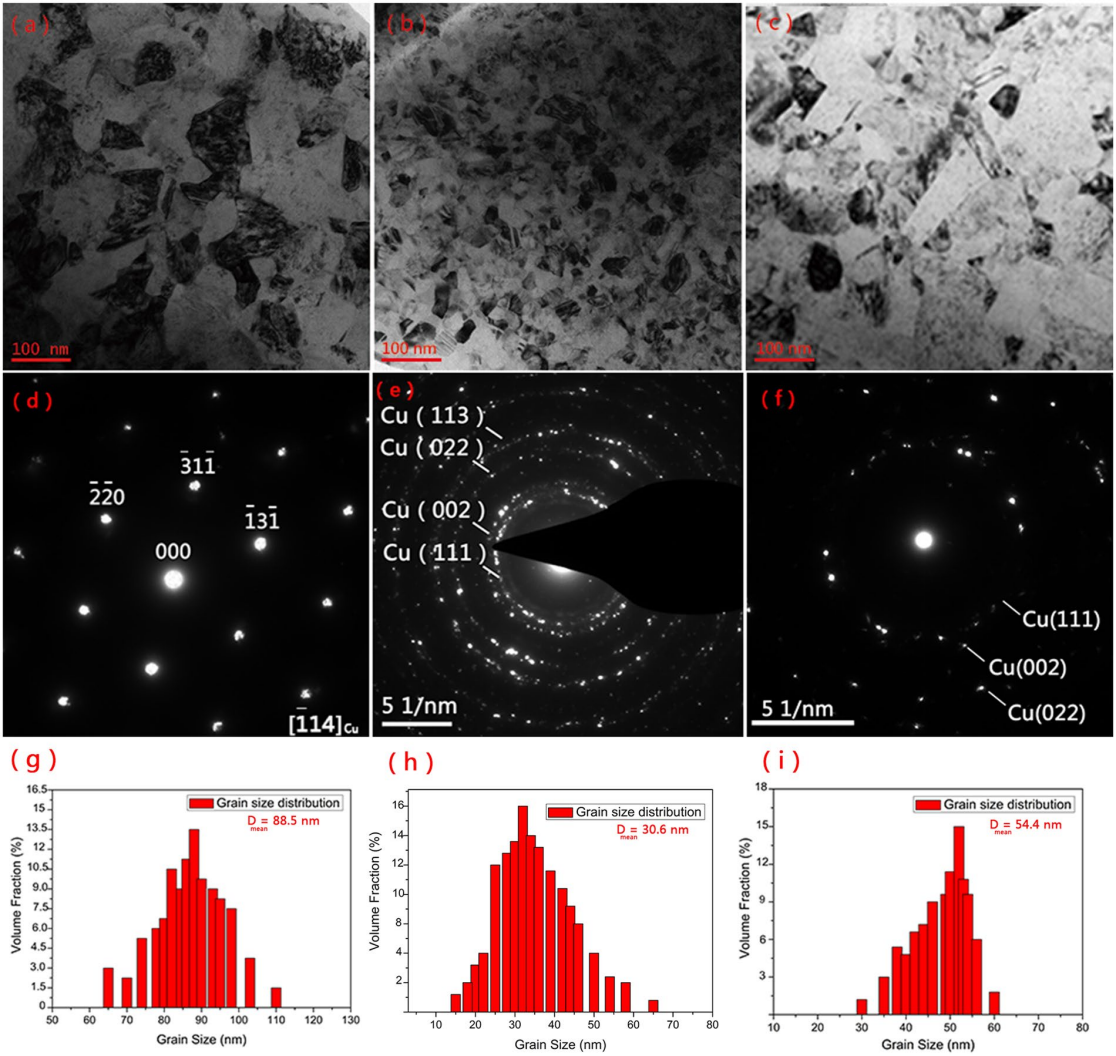
The TEM (**a**–**c**) and the corresponding SEAD (**d**–**f**) images and grain size distribution (**g**–**i**) of pure Cu, RGO/Cu-5 and RGO/Cu-7, respectively.

Figure 6 showed that the presence of RGO has great effects on crystallite size and microstrain of RGO/Cu composites. It can be calculated that the crystallite size of pure Cu and RGO/Cu composites (RGO/Cu-1, RGO/Cu-3, RGO/Cu-5, RGO/Cu-6, RGO/Cu-7) is 103 nm, 73 nm, 57 nm, 35 nm, 55 nm and 66 nm, respectively. Obviously, the crystallite size of RGO/Cu composites firstly decreased with the increase of GO content in the electrolyte from 1 mg/L to 5 mg/L, then increased with further adding GO from 5 mg/L to 7 mg/L. Actually, proper content of graphene can effectively restrict the growth of metal crystallites^2, 14^. This could be ascribed to two reasons: (1) graphene in the matrix would precipitate at the grain boundaries during solidification and act as a barrier for crystals growth, and (2) graphene can provide more supporting sites for metal nucleation because of their high specific surface area. If nucleation sites and/or nucleation rate increased, the mean crystallite size would correspondingly decreased^45^. Apparently, the decreased grain size contributed to the increase of nanohardness because the volume fraction of grain boundary increased, and thus dislocation motion could be blocked at the sites of RGO and resistance to localized plastic deformation^6, 14^. However, by further increasing of GO in the electrolyte (>5 mg/L), the grain size of RGO/Cu composites increased (Figs 6 and 7) and nanohardness decreased significantly (Fig. 5). This result was mainly because of RGO aggregation in the process of co-deposition, which might restrict matrix materials to flow into the agglomerates. Besides that, higher GO concentration in the bath (more than 5 mg/L in our GO/Cu^2+^ system) led to destabilization of electrolyte due to positively charged copper ions could combine with negatively charged GO sheet, and hence introducing the agglomeration in the mixture solution^22^. This serious aggregation could cause large porosity in the composites and weaken the mechanical performance^6^. Moreover, based on the Zener pinning, the average grain size can be calculated^46^:

5 $$D=\frac{k\,\ast \,r}{{f}^{n}}$$

The term k is a proportional dimensionless constant, f is the volume fraction of the second phase and r is the mean reinforcement radius. Obviously, the refinement effect is largely dependent on the size of the RGO, being directly proportional to their size. Therefore, by increasing the amount of agglomerates, the real size of the reinforcements is increased resulting in larger final grain sizes. Additionally, Fig. 7(a–c) showed the typical bright-field images of pure Cu, RGO/Cu-5 and RGO/Cu-7, respectively. The Fig. 7(d–f) and (g–i) revealed their corresponding electron diffraction pattern (SEAD) and grain size distribution. It was clear to find that the grain size (the mean grain size of Cu, RGO/Cu-5 and RGO/Cu-7 is 88.5 nm, 30.6 nm and 54.4 nm) decreased obviously because of the incorporation of RGO reinforcement but increased when the addition of exceed GO (RGO/Cu-7), which was consistent with the results of XRD.

Besides that, there is a great difference of thermal expansion coefficients (CTE) exists between RGO and Cu matrix (CTE for graphene is −6 × 10^−6^ K^−1^ at 300 K, Cu is 24 × 10^−6^ K^−1^). This mismatch in RGO/Cu specimens could give rise to amount of dislocations, which promoted the strengthening of the metal matrix^39, 40^.

Additionally, Fig. 6 showed that the microstrain increased with an increase in the additive amount of GO. The addition of RGO in Cu matrix resulted in compressive micro-strain, which further led to distortion of metal lattice^42, 47^. Here, the lattice parameter of Cu matrix composites reinforced with RGO was calculated based on cohen’s method^42^. According to the Bragg law:

6 $$\lambda =2d\,\sin \,\theta $$

where λ is the wavelength of the X-radiation used (λ = 0.154 nm), d is the interplanar spacing and θ is the Bragg angle.

By taking the logarithms and differentiating Eq. (6),

7 $$\frac{{{\rm{\Delta }}\sin }^{2}\theta }{{\sin }^{2}\theta }=\frac{-\,2{\rm{\Delta }}d}{d}=-\,2K{\cos }^{2}\theta $$

where Δd/d is relative error in d, which approaches zero as the diffraction angle θ approaches 90°, and K is a constant.

Equation (7) can be converted to

8 $${{\rm{\Delta }}\sin }^{2}\theta =-\,2K{\cos }^{2}\theta {\sin }^{2}\theta =D{\sin }^{2}2\theta $$

where D is the drift constant.

9 $${{\rm{\Delta }}\sin }^{2}\theta ={\sin }^{2}\theta ({\rm{observed}})-{\sin }^{2}\theta ({\rm{true}})$$

For the cubic system, the Δsin2θ can be added to the Eq. (6)^48^

10 $${\sin }^{2}\theta =\frac{{\lambda }^{2}}{4{a}_{0}^{2}}({{\rm{h}}}^{2}+{{\rm{k}}}^{2}+{{\rm{l}}}^{2})+{{\rm{Dsin}}}^{2}2\theta $$

The Eq. (10) gets reduced to:

11 $${\sin }^{2}\theta =C\alpha +D\delta $$

12 $$C={\lambda }^{2}/4{\alpha }_{0}^{2},$$

13 $$\alpha ={h}^{2}+{k}^{2}+{l}^{2}$$

14 $$\delta ={\sin }^{2}2\theta $$

According to the method of least squares, the most probable values of the parameters C and D are those make the sum of squares $({\sin }^{2}{\theta }_{i}-C{\alpha }_{i}-{\rm{D}}{\delta }_{i})$ a minimum:

15 $$f(C,{\rm{D}})=\sum _{i}{({\sin }^{2}{\theta }_{i}-C{\alpha }_{i}-{\rm{D}}{\delta }_{i})}^{2}$$

16 $$\frac{\partial f(C,{\rm{D}})}{\partial A}=\frac{\partial f(C,{\rm{D}})}{\partial D}=0$$

17 $$\sum _{i}{\sin }^{2}{\theta }_{i}{\alpha }_{i}=C\sum _{i}{\alpha }_{i}^{2}+D\sum _{i}{\alpha }_{i}{\sin }^{2}2{\theta }_{i}$$

18 $$\sum _{i}{\sin }^{2}2{\theta }_{i}\,\ast \,{\sin }^{2}{\theta }_{i}=C\sum _{i}{\alpha }_{i}{\sin }^{2}2{\theta }_{i}+D\sum _{i}{\sin }^{4}2{\theta }_{{\rm{i}}}$$

Obviously, the lattice parameter (α_0_) can be determined from Eq. (12). α come from the corresponding peaks (h k l). The lattice parameters were given in Table 1.

**Table 1:** The lattice parameter calculated from XRD for RGO/Cu composites and pure Cu sample.

composites	2θ	(h, k, l)	Sin 2θ	a_0_ (*Å*)
Pure Cu	43.35	(111)	0.6864	3.6148
	50.45	(200)	0.7710	
	74.15	(220)	0.9619	
RGO/Cu-1	43.460	(111)	0.6878	3.6114
	50.600	(200)	0.7727	
	74.340	(220)	0.9628	
RGO/Cu-3	43.519	(111)	0.6885	3.6103
	50.680	(200)	0.7736	
	74.435	(220)	0.9633	
RGO/Cu-5	43.550	(111)	0.6889	3.6091
	50.689	(200)	0.7737	
	74.47	(220)	0.9634	
RGO/Cu-6	43.603	(111)	0.6896	3.6078
	50.701	(200)	0.7739	
	74.52	(220)	0.9637	
RGO/Cu-7	43.625	(111)	0.6899	3.6045
	50.74	(200)	0.7743	
	74.58	(220)	0.9640	

While the above calculated lattice parameter of Cu was 3.6148 *Å*, which was excellent consistence with previous reports (3.615 *Å*)^49^. It proved that the cohen’s method was feasible and reliable used in graphene reinforced metal materials. Moreover, the lattice parameter of RGO/Cu samples between RGO/Cu-1 and RGO/Cu-7 decreased from 3.6114 down to 3.6045 with increasing RGO content. This was mainly because compressive micro-strains arising from RGO addition caused lattice shortening of Cu matrix^42^. Furthermore, with the increasing RGO content, the shortening of lattice parameter resulted in the enhancement of interaction, which then led to the increase of the hardness of RGO/Cu composites^50^.

Moreover, the dislocation density of RGO/Cu specimens can be further investigated by XRD. Figure S4 exhibited a typical dislocation in the RGO/Cu composites. According to the Williamson relationship, the quantitative dislocation densities can be calculated in terms of the fitted results of domian size^51, 52^.

19 $$\rho =\frac{2\sqrt{3}}{|\overrightarrow{b}|}{\frac{\langle {\varepsilon }^{2}\rangle }{D}}^{1/2}$$

20 $$|\overrightarrow{b}|=\frac{a}{2}\sqrt{{u}^{2}+{v}^{2}+{w}^{2}}$$

where ρ is the dislocation density, ε is the microstrain of the composites and a is the unit cell edge length which have been calculated in Table 1 and Fig. 6, 〈ε^2^〉 is the weighted average of ε^2^, D is the domain size and |b| is the mold of Burgers vector of Cu, and u, v, w are the indices of crystal along the gliding orientation. Therefore, the corresponding estimation dislocation density generated by incorporation of graphene (RGO/Cu-1, RGO/Cu-3, RGO/Cu-5, RGO/Cu-6, RGO/Cu-7) is around 3.68 × 10^−4^ nm^−2^, 6.06 × 10^−4^ nm^−2^, 1.156 × 10^−3^ nm^−2^, 7.62 × 10^−4^ nm^−2^ and 6.51 × 10^−4^ nm^−2^, respectively).

Orowan looping strengthening is another strengthening mechanisms which is about fine precipitates strengthening^41^. This mechanism can be ascribed to hampering movements of dislocation by the nanometer scales RGO at the grain boundaries. Generally, the bending of these dislocations existed around the RGO, where the dislocation loops formed. In fact, several literatures have proved that Orowan looping strengthening is an important strengthening mechanisms for graphene reinforced metal composites which is about fine precipitates strengthening^14, 40^. Unfortunately, in our case, the Orowan looping strengthening effect may not be very significant because the size of RGO in the Cu matrix was mainly in micro and sub-micro scale (Fig. 2).

**Figure 8:**
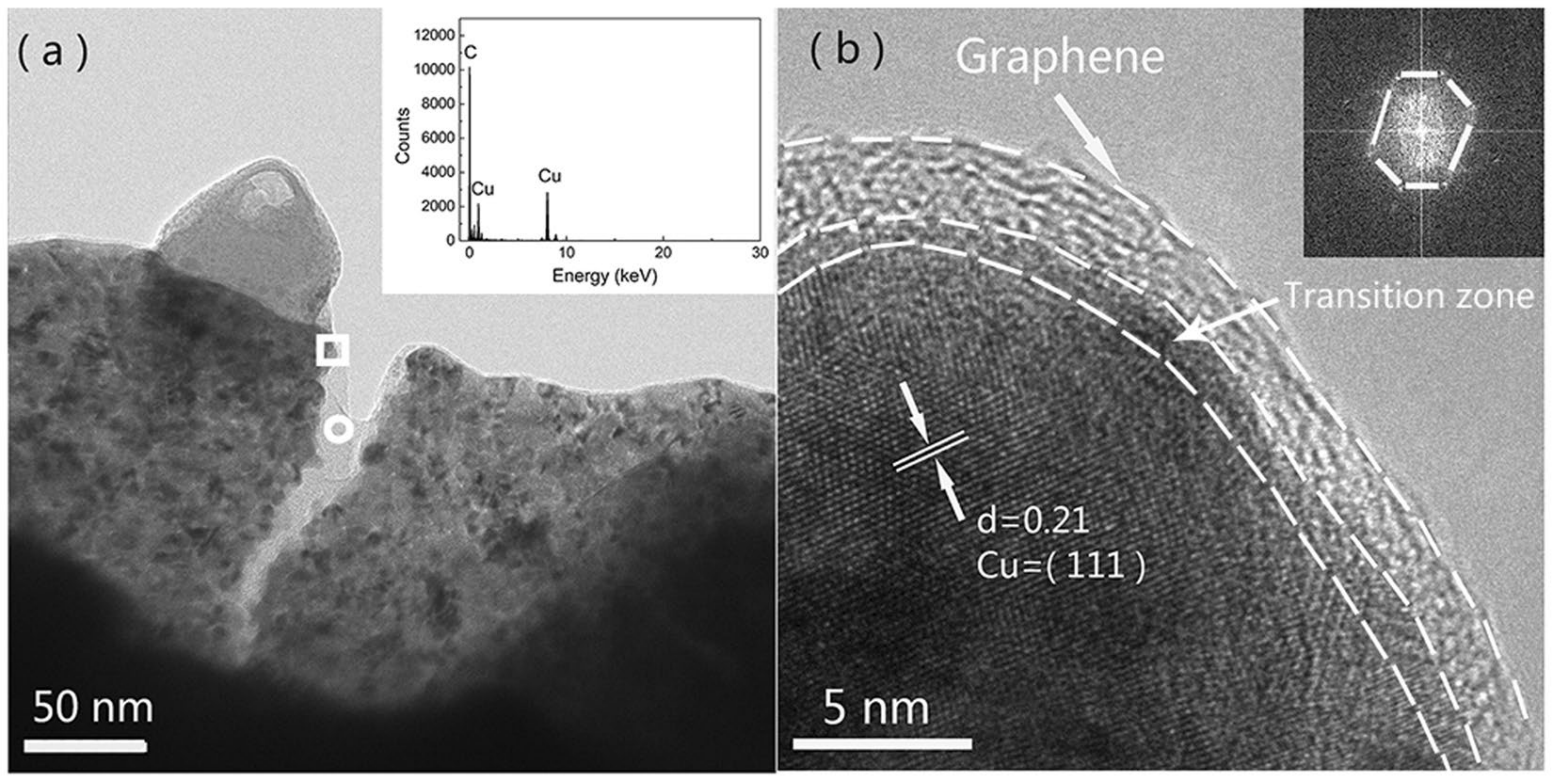
(**a**) Low magnification TEM image of the RGO/Cu composites and corresponding EDS analysis (inset of Fig. 2d) for the element distribution at marked circle region. (**b**) High magnification image taken from the image of marked rectangular area of Fig. 8b, the inset (FFT pattern) showed that RGO has several crystal domains.

Additionally, load transfer also played an important role in improving the hardness of graphene-reinforced composites. Shin, S.E^5^ indicated that when graphene-reinforced metal composites was loaded, the matrix was strained and then the strained matrix could transfer the load to reinforcements (graphene) by means of shear stresses. Besides that, graphene which has ultra-high intrinsic strength (125 GPa) was expected to undergo a substantial part of the mechanical load in the composites based on a good interfacial bonding^6, 40, 53^. Figure 8a showed the TEM image of RGO/Cu-5 composite. It can be seen that the large transparent RGO in the form of fewer layers existed in the copper matrix with a typical crumpled structure. Additionally, the EDS on marked circle box (the inset image in Fig. 8a) revealed that the transparent and ripple part was mainly consisted of C element. Figure 8b showed the high-resolution TEM image of the rectangular box of Fig. 8a. It revealed that the lattice distance was 2.1 *Å*, which was agreement with the distance of (1 1 1) lattice spacing of Cu crystal. The inset image of Fig. 8b showed the fast Fourier transform (FFT) result of the marked dashed line area. It exhibited a clear hexagonal and crystalline structure, which represented the periodic carbon structure of graphene^54^. Furthermore, it was clear that RGO was embedded into the Cu matrix and there was a transition zone exist between RGO and Cu matrix. Cavities and reaction products were not observed in this transition zone, indicating that there was a strong interfacial bonding between the RGO and Cu matrix and thus the load can effective transfer from Cu matrix to the RGO.

Furthermore, Mina Park^55^ calculated the chemical interaction between Cu and functionalized CNT surfaces based on the density functional theory method. They revealed that the oxygen-containing functional group could significantly enhance the interfacial bonding between Cu and CNTs due to electron exchange between Cu and carbon atoms. In our case, FTIR and XPS (Fig. 3b,c and d) showed that there was still parts of residual functional groups existed in the RGO after electroless co-deposition, and the characteristic peaks of FTIR at 550–620 cm^−1^ can be attributed to the Cu-O stretching vibration^40^. It suggested that the oxygen-containing functional groups of RGO might interact with Cu^2+^ ions (open the epoxide ring) and formed chemical bonds between RGO and Cu^2+^ ions^40^. Obviously, this interaction was beneficial to load transfer from Cu matrix to RGOs.

## Conclusion

In summary, we successfully prepared the Cu matrix composite reinforced by RGO through electroless co-deposition and investigated its nanohardness under different GO contents in electrolyte. XRD analysis revealed a decrease in matrix grain size and no oxides/carbides formatted in the synthesis process. The calculation of XRD results proved that the addition of RGO in the Cu matrix could lead to the compressive micro-strain, which further caused lattice shortening due to the presence of RGOs. Compared to pure Cu, the incorporation of RGOs in Cu matrix could remarkably improve their nanohardness. The improvement was gradually enhanced with increasing GO contents in the electrolyte and an optimal result was obtained at 5 mg/L of GO in the electrolyte. The strengthening mechanism could be attributed to dislocation generation because of the mismatch in coefficient of thermal expansion, grain refinement, Orowan looping and load transfer from matrix to reinforcement. Obviously, the incorporation of graphene in the composites played an important role in the enhanced mechanical performance of the RGO/Cu materials. We believe that such a simple and low-cost method provides a new pathway for fabrication of graphene-reinforced metal composites.

## Methods

### Material fabrication

The RGO/Cu composites synthesized here were deposited on a carbon-steel substrate by an electroless process in a copper sulfate electrolyte with the following composition: 8 g/L CuSO_4_·5H_2_O, 0.5 g/L NiSO_4_·6H_2_O, 20 g/L Na_3_C_6_H_5_O_7_·2H_2_O, 30 g/L H_3_BO_3_ and 40 g/L NaH_2_PO_2_·H_2_O. Certain amount of GO solution was achieved by ultrasonication (GO was purchased from Nanjing XFNANO Materials Tech Co., Ltd.). NaH_2_PO_2_ is used as reducing agent in electroless deposition. The NiSO_4_•6H_2_O (Ni^2+^) is used as catalyst to catalyze the hypophosphite oxidation enabling continuous copper deposition because catalytic activities of Ni is higher than Cu^56, 57^. Firstly, the carbon-steel substrate should be polished with SiC abrasive paper, and then performed two pretreatments: alkaline degreasing (10% NaOH) and acid activation (10% H_2_SO_4_). After that, the specimen was sensitized by SnCl_2_ solution and then activated in PdCl_2_ solution prior to plating. In the sensitization process, Sn^2+^ ions were absorbed on the surface of the substrate, which could facilitate attracting of Pd^2+^ ions and worked as reducing agents for Pd deposition^58, 59^. This process can be represented by the following equation:

21 $$S{n}^{2+}+P{d}^{2+}\to S{n}^{4+}+P{d}^{0}$$

The presence of Sn^4+^ can stabilize the Pd metals via a strong Sn^4+^ adsorption and then very small Pd particles are obtained. Generally, the adhered Pd particles was used as activators and render catalytic sites for the nucleation of metals^60, 61^. Then, the GO suspension was slowly dispersed into the electrolyte when temperature reach at 50–55 °C, followed by adding the reducing agent (NaH_2_PO_2_) to the mixture at 65–70 °C.

When the activated substrate was immersed vertically in the plating solution, the deposition process (Eqs 22 and 23) commenced spontaneously. The Pd^0^ acted as catalyst and GO/Cu^2+^ will be reduced to metallic RGO/Cu by NaH_2_PO_2_, and simultaneously the metallic Pd^0^ oxidized to Pd^2+^ during the subsequent Cu deposition process. As the reaction continues, the metallic RGO/Cu could be deposited and become denser and thicker on the substrate.

22 $$Pd+C{u}^{2+}\to P{{\rm{d}}}^{2+}+C{\rm{u}}$$

23 $${[C{\rm{u}}/\mathrm{GO}]}^{2+}+{H}_{2}P{O}_{2}^{-}+{H}_{2}O\to {H}_{2}P{O}_{3}^{-}+{H}_{2}+RGO+C{u}^{0}\downarrow $$

Additionally, the pH of electrolyte need to maintain at around 9.0 through addition of NaOH, and a magnetic stirrer was used to maintain homogenous of the electrolyte during the whole electroless process. Pure Cu samples were also prepared using the electroless deposition under the same synthesis conditions for comparison. The relative density of RGO/Cu composites was added in Supporting information (Figure S5).

### Microstructural characterization

The morphology of samples was characterized by atomic force microscopy (AFM, AXIS ULTRA DLD), scanning electron microscopy (SEM, JEOL, 7600 F) and transmission electron microscope (TEM, JEOL, 2100 F). Raman spectroscopy (Senterra R200-L), Fourier transform infrared spectra (FTIR, EQUINOX55 spectrometer) and X-ray photoelectron spectroscopy (XPS, AXIS Ultra DLD, Kratos) was used to determine the microstructure of graphene. The crystal structures of the RGO/Cu composites were investigated by X-ray powder diffraction (XRD, RINT-2000, Rigaku). The samples were scanned with a scanning rate of 5°/min.

### Nanoindentation tests

The nanohardness of RGO/Cu samples were performed by Nanoindenter (G200, Agilent) equipped with the Berkovich indenter. All the nanoindentation tests were conducted under the continuous stiffness measurement (CSM) model. CSM offers the direct measurement of dynamic contact stiffness at every data point acquired during the indentation experiment. The prescribed maximum displacement was 3000 nm and 5 indents with a space of 25 um were made on each sample.

## Electronic supplementary material

Supporting Information
