# Supplementary material for: Microstructure and nanoindentation behavior of Cu composites reinforced with graphene nanoplatelets by electroless co-deposition technique
Source: Sci Rep. 2017 May 2;7:1338. doi: 10.1038/s41598-017-01439-3 (PMC5431065; doi:10.1038/s41598-017-01439-3)
Supplement: Supplementary file 1 — Supporting Information [file 41598_2017_1439_MOESM1_ESM.pdf]

Supporting Information

Microstructure and nanoindentation behavior of Cu composites reinforced with graphene nanoplatelets by electroless co-deposition technique

Qi Zhang[a, b], Zhenbo Qin [a, b], Qin Luo [a, b], Zhong Wu[*, a, b], Lei Liu[*, a, b],

Bin Shen[a, b], Wenbin Hu[c]

[a]*State Key Laboratory of Metal Matrix Composites, Shanghai Jiao Tong University, Shanghai, 200240, China*

[b]*Collaborative Innovation Center for Advanced Ship and deep-Sea Exploration, Shanghai, 200240, China*

[c]*School of Material Science and Engineering, Tianjin University, Tianjin, 300072, China*

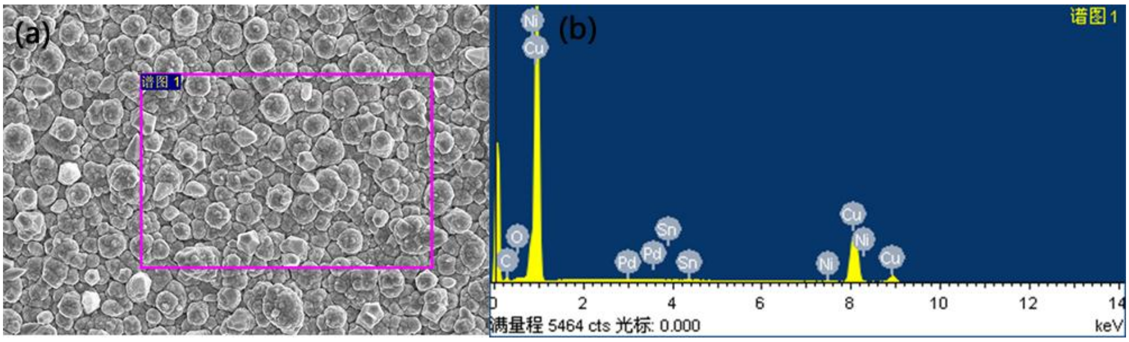

Figure S1. SEM micrograph of (a) RGO/Cu composites; (b) represent the corresponding EDS profile analysis of the surface shown in the micrograph.

Table S1. EDS analysis of RGO/Cu composites

元素	重量百分比	原子百分比
C K	12.15	41.66
O K	0.69	1.77
Ni K	1.38	0.97
Cu L	85.78	55.60
Pd L	0.00	0.00
Sn L	0.00	0.00
总量	100.00	

* Corresponding author. Tel.: +86 02134203812; Fax: +86 021 34202749
E-mail: wuzhong2319@163.com.

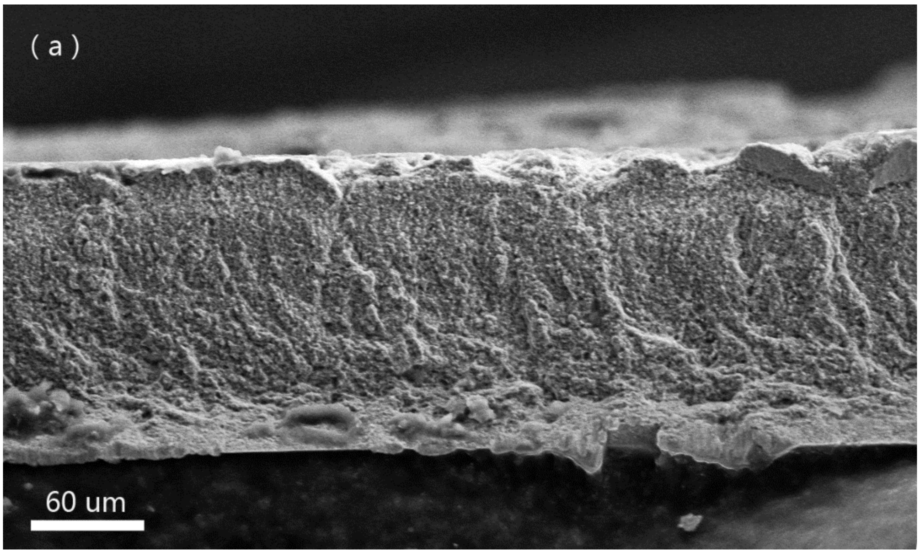

Figure S2. The cross section of a typical RGO/Cu composite.

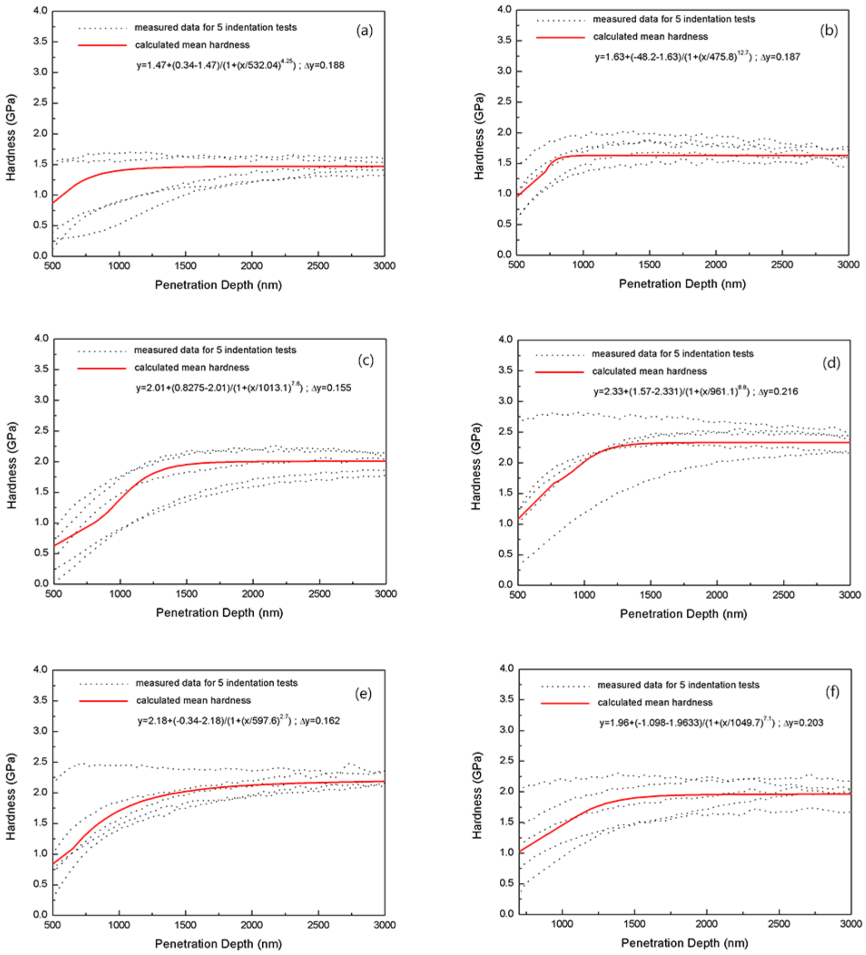

Figure S3. Hardness as function of indentation depth: (a) pure Cu; (b) RGO/Cu-1; (c) RGO/Cu-3; (d) RGO/Cu-5; (e) RGO/Cu-6; (f) RGO/Cu-7 (The dotted line was the experimental result of nanoindentation, and the solid line represented the fitted result of the experimental hardness data according to a typical Logistic analysis). These fitted lines were used for the curves of Figure 5a.

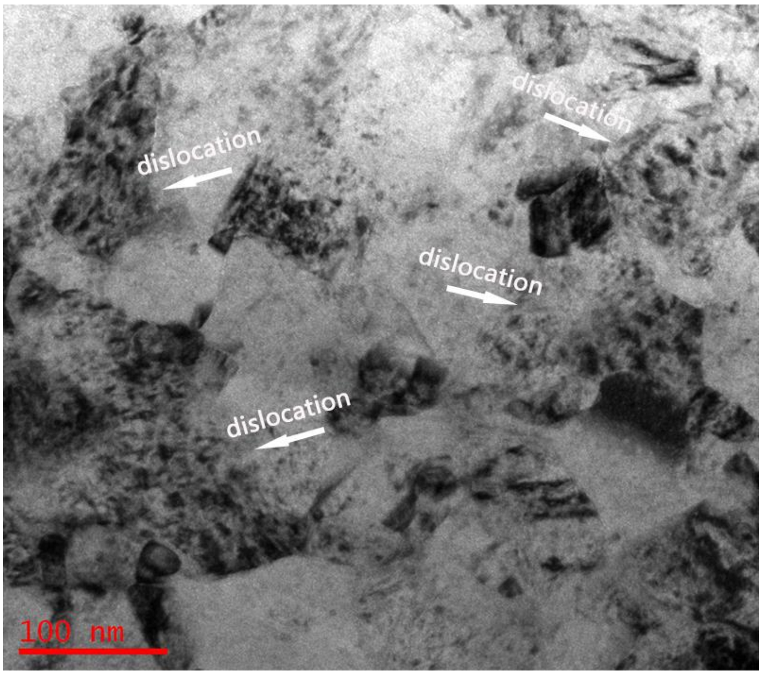

Figure S4. TEM image of the typical dislocation in RGO/Cu composite.

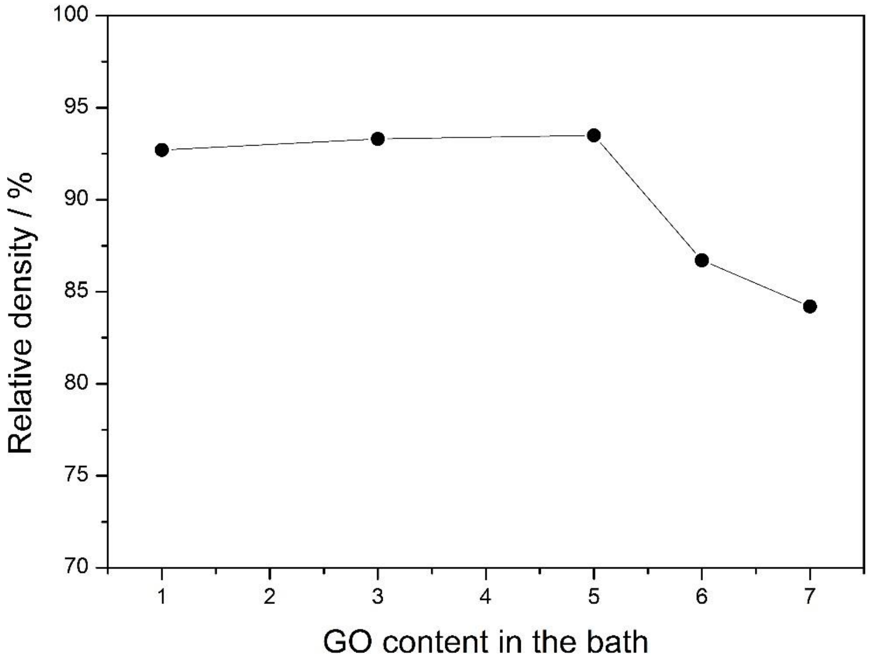

Figure S5. The relative density of RGO/Cu composites at different GO loading in bath.
